# Total Coliform and Generic *E. coli* Levels, and *Salmonella* Presence in Eight Experimental Aquaponics and Hydroponics Systems: A Brief Report Highlighting Exploratory Data

**DOI:** 10.3390/horticulturae6030042

**Published:** 2020-07-30

**Authors:** Daniel L. Weller, Lauren Saylor, Paula Turkon

**Affiliations:** 1 Department of Environmental and Forest Biology, State University of New York, Environmental Science and Forestry, Syracuse, NY 13210, USA; 2 Department of Biostatistics and Environmental Health, University of Rochester Medical Center, Rochester, NY 14642, USA; 3 Department of Environmental Studies and Sciences, Ithaca College, Ithaca, NY 14850, USA

**Keywords:** aquaponics, salmonella, *Escherichia coli* (*E. coli*), hydroponics, food safety, produce safety

## Abstract

Although many studies have investigated foodborne pathogen prevalence in conventional produce production environments, relatively few have investigated prevalence in aquaponics and hydroponics systems. This study sought to address this knowledge gap by enumerating total coliform and generic *E. coli* levels, and testing for *Salmonella* presence in circulating water samples collected from five hydroponic systems and three aquaponic systems (No. of samples = 79). While total coliform levels ranged between 6.3 Most Probable Number (MPN)/100-mL and the upper limit of detection (2496 MPN/100-mL), only three samples had detectable levels of *E. coli* and no samples had detectable levels of *Salmonella*. Of the three *E. coli* positive samples, two samples had just one MPN of *E. coli*/100-mL while the third had 53.9 MPN of *E. coli*/100-mL. While the sample size reported here was small and site selection was not randomized, this study adds key data on the microbial quality of aquaponics and hydroponics systems to the literature. Moreover, these data suggest that contamination in these systems occurs at relatively low-levels, and that future studies are needed to more fully explore when and how microbial contamination of aquaponics and hydroponic systems is likely to occur.

## 1. Introduction

Multiple foodborne disease outbreaks and recalls have been traced back to preharvest contamination of fresh produce (e.g., [1–3]). For example, several recent *Escherichia coli (E. coli)* O157:H7 outbreaks linked to romaine lettuce have been traced back to the use of contaminated irrigation water [4,5]; the 2018 outbreak linked to lettuce grown in Yuma, AZ resulted in 210 illnesses, 96 hospitalizations, and 5 deaths across 36 states [5]. Due to the substantial public health and economic burden of produce-associated outbreaks, preventing preharvest contamination is a priority for academic, government, and industry stakeholders [6,7]. Indeed, multiple studies have been conducted to investigate the prevalence, distribution, and dispersal of foodborne pathogens in and between farm and farm-adjacent environments [8–10]. For instance, a series of studies conducted in California and New York examined the transfer of *E. coli* from wildlife feces to preharvest lettuce via splash during irrigation [11–14]. Similar field studies have been conducted to examine pathogen survival in and transfer to produce from soil, irrigation water, and other environmental sources [15–21]. However, the majority of research has focused on soil-based field and greenhouse environments, and there is limited data on food safety hazards in soil-free production environments, such as hydroponics (i.e., production of plants in a liquid media instead of soil) and aquaponics (i.e., system that combines aquaculture [raising of fish or other seafood] with hydroponics) systems. Indeed, a recent topical summit that gathered academic, industry and government experts identified the need for additional data on hazards in hydroponic and aquaponics systems, and specialized resources for growers who utilize these systems [22].

Although relatively few studies have investigated the prevalence and distribution of food safety hazards in aquaponics systems, these systems are of increasing interest as a way to address food sustainability and security needs. Studies conducted in conventional and greenhouse production environments, and fish supply chains indicate that multiple pathways exist for the introduction of foodborne pathogens into hydroponic and aquaponic systems, including, but not limited to, fish feed, fish waste, the system’s water, and the vegetable seeds [3,23–32]. Due to the limited number of studies that investigated food safety hazards in aquaponics or hydroponics systems [27,32–35], additional prevalence data are needed to fully characterize and manage food safety hazards associated with various aquaponics and hydroponics inputs. This need is particularly pressing, since the data that currently exist vary substantially between studies. For example, a study that surveyed pathogen levels in six experimental systems isolated Shiga-toxin producing *E. coli* but not *Listeria monocytogenes* or *Salmonella* from water, fish feces, and produce root samples [27]. Conversely, a study that sampled commercial and backyard aquaponics systems in Hawaii failed to detect *E. coli* O157:H7 or *Salmonella* in 510 fish feed, fish, and produce samples [36]. However, an unpublished study from the University of Minnesota did detect *Listeria* in aquaponically, and hydroponically-grown lettuce at retail [37]. The present study was conducted to generate data on the levels and prevalence of microbial contaminants in three aquaponics and five hydroponics systems in New York, to help address this knowledge gap and to generate preliminary data on which future studies can build. Since past studies have shown that water is a key pathway for the introduction of foodborne pathogens into production environments, and can, directly and indirectly, transfer pathogens to produce [11,13,38–42], the current study specifically focused on characterizing microbial contamination of water in the eight aquaponics and hydroponics systems sampled here.

## 2. Methods

### Systems.

Water samples were collected from eight experimental systems in Ithaca, New York, including 3 aquaponics and 5 hydroponic systems (Table 1). While all systems were essentially similar in overall design (e.g., use of municipal water), systems differed in size, temperature, and potential for food safety contamination (e.g., presence of foot traffic; open-air; Table 1). Four of the experimental systems (three hydroponic and one koi-based aquaponic system at Location C) were located in a greenhouse with temperature maintained at 16–29 °C, and with limited public access and no food safety protocols (e.g., regarding handwashing prior to interacting with the systems). The fifth system (hydroponics system at Location T) was an open-air system in a large dining establishment providing several hundred individuals access to the system on a daily basis, and resulting in temperature staying at approximately 21–25 °C. The sixth system (a catfish-based aquaponics system) was in an un-insulated greenhouse with minimal foot traffic (air temperature ranged between −1 and 37; Location H), while the remaining aquaponics (tilapia-based) and hydroponics systems were in a heated greenhouse (air temperature approximately 19–23 °C), utilized water heaters (approximately 19–27 °C), and had limited public access.

### Sampling and Bacterial Assays.

Water samples were collected weekly from seven systems for ten weeks, and from one system for nine weeks; the latter was enrolled after the first set of samples were collected (No. of samples total = 79). Samples were collected between January and March 2018. At each sampling 100-mL of water was collected from the reservoir used to collect water prior to recirculation. Samples were collected by submerging the sampling bottle 15 cm below the water surface. Samples were held on ice and processed within 1 h of sample collection. Total coliform and *E. coli* (a fecal indicator bacteria [FIB]) concentrations were enumerated in 100-mL of the sample using the IDEXX Colilert Quanti-tray 2000 per manufacturer’s instructions and as previously described [43,44]. While there are limitations to this method (e.g., inability to detect viable but non-culturable [VBNC] bacteria), this approach is consistent with current produce safety water quality standards in the United States. Indeed, the IDEXX Colilert Quanti-tray 2000 is an approved method for conducting *E. coli* testing as proscribed in Food Safety Modernization Act’s Produce Safety Rule [45]. Gloves were changed between each sample collection, and all sampling equipment, including the gloved hands, were sterilized with 70% ethanol. Positive (B-strain *E. coli*) and negative (sterile water plus Colilert reagent) controls were run in parallel with each sampling set; all negative samples had <1 most probable number (MPN) per 100-mL (this was the lower limit of detection [LOD]), while all positive samples had >2419.6 MPN/100-mL (the upper LOD). For one system per week, an additional 25-mL was used for *Salmonella* detection (i.e., 9 samples were tested for *Salmonella* in the study reported here). The 25-mL samples collected from systems with detectable levels of *E. coli* were preferentially selected for *Salmonella* testing. During weeks when all samples were below the lower limit of detection for *E. coli*, one sample was randomly selected for *Salmonella* detection. Random selection was performed to ensure that at least one sample per system was sent for *Salmonella* testing. Samples were shipped on ice to Eurofins Scientific Laboratories, who performed the analyses for *Salmonella* detection using AOAC method 2003.09 [46]. This method uses the BAX assay as a PCR-screen followed by culture-confirmation of any PCR-positive samples [46]; past studies have used similar methods for detecting *Salmonella* in water [43,44]. Since a PCR-screen is performed before culture-confirmation, the presence of dead and viable but non-culturable (VBNC) *Salmonella* is possible using this method. However, dead and VBNC cannot be distinguished using this approach. All *E. coli* and coliforms data are available in Supplemental Materials-Raw Data.

## 3. Results and Discussion

Of the 79 samples collected, 100% tested positive for coliforms. Specifically, 61 samples were above the upper LOD for the IDEXX Colilert Quanti-Tray 2000 (2419.6 MPN/100-mL). The 16 samples below the upper LOD ranged between 6.3 and 1986.3 MPN of total coliforms/100-mL (mean = 1024.7 MPN/100-mL). The five samples with the lowest total coliform levels all came from H-A (Table 2). H-A thus had the lowest level of total coliforms of the eight systems sampled and was also the only site without public access or foot traffic. Ninety-six percent (76/79) of samples were below the LOD for generic *E. coli* (Supplemental Materials-Raw Data; 95% Confidence Interval [95% CI] = 0–3.7 MPN/100-mL; Table 2). Of the three samples that were above the LOD (1 MPN/100-mL) for generic *E. coli*, two samples had 1 MPN of *E. coli*/100-mL (95% CI = 0.1–5.5 MPN of *E. coli*/100-mL) and were collected from systems T-A and C-B. The third sample that was above the LOD was collected from system C-C and had 53.9 MPN of *E. coli*/100-mL (95% CI = 40.5–69.7 MPN of *E. coli*/100-mL; Table 2). Of the three samples that were above the LOD, each came from separate systems indicating low-level contamination in multiple systems (Table 2). All nine samples sent for *Salmonella* testing were below the limit of detection for the test (LOD = 1/25-mL), indicating the probable absence of *Salmonella* in the system at the time of sample collection.

Due to the limited number of samples positive for generic *E. coli* or *Salmonella*, and below the upper LOD for total coliforms no statistical analyses could be performed here. While the limited sample size (N = 79 for samples where FIBs were enumerated, and N = 9 for samples where pathogen testing was performed) is a limitation of the study reported here, preliminary studies, including negative results, provide key data on which future studies can build. Indeed, despite the presence of potential risk factors for microbial contamination (e.g., public access, absence of handwashing protocols), fecal contamination (as indicated by *E. coli*, a fecal indicator bacteria) was detected in 4% of samples. Future research on pathogen prevalence in aquaponics and hydroponics systems can use these data to guide sample size calculations for their studies. For instance, based on the 4% prevalence found here, a future study that wanted to estimate *E. coli* prevalence in aquaponics and hydroponics systems with 95% confidence and 2% precision, would need to collect 188 samples (sample size estimation performed using formula in [47]). If a higher precision or confidence is desired then the necessary sample size would increase (1% precision increases sample size to 753, 97.5% confidence increases sample size to 224).

Despite the small sample size of the study reported here, the results are consistent with the findings of several past studies [32–34]. For example, a study that collected basil, lettuce, barramundi and water from 6 laboratory aquaponics-systems over 118 days failed to detect generic *E. coli, E. coli* O157:H7, or *Salmonella* in any of the samples [34]. This previous study, like the study reported here, found similar levels of coliforms (between 13 and 1820 CFU/100-g; calculated using data reported in the study; [34]). Also similar to the study reported here, a study that assessed microbial contamination on lettuce and water samples collected from two Puerto Rican hydroponic facilities failed to detect *Salmonella* [32].

Interestingly, the low levels of microbial contamination reported here and in previous aquaponics and hydroponics studies, contrasts to the substantially higher levels of microbial contamination reported by studies conducted in conventional production environments. For example, all 181 agricultural water samples collected from conventional produce farm environments in New York in a 2017 study had detectable levels *E. coli* (Mean = 181.5 MPN/100-mL; Range = 18.5 to >2419.6 MPN/100-mL), while 44% (80/181) of samples were *Salmonella*-positive. The two agricultural water studies are not unique as multiple studies conducted in Arizona [44], Belgium [10], Florida [48], New York [40,43], South Africa [49], Spain [50], and Virginia [51] also reported higher fecal indicator bacterial levels and higher pathogen prevalence than the present study. While this may suggest a lower likelihood of food safety hazards in aquaponic and hydroponic systems compared to conventional agriculture, additional studies are needed to directly compare food safety hazards in these to conventional environments. Despite this need for additional research, the results of this and other aquaponics/hydroponics food safety studies indicate that contamination in aquaponics/hydroponics systems occurs at low-levels [32–34], and that large sample sizes are needed in future observational studies to fully characterize pathogen prevalence in these systems.

## Figures and Tables

**Table 1.** Characteristics of the eight systems sampled here.

Location	System		Public Access		Temperature Range (°C)		Crop	Fish

	ID	Type ^a^	Who	Frequency	Air	Water		
C	A	Hydroponic	Researchers, Students	Frequent	16–29	16–29	Strawberries	-
	B	Hydroponic	Researchers, Students	Frequent	16–29	60–29	Strawberries	-
	C	Hydroponic	Researchers, Students	Frequent	16–29	16–29	Strawberries	-
	D	Aquaponic	Researchers, Students	Frequent	16–29	16–29	Strawberries	50 Koi
H	A	Aquaponic	None	Infrequent	−1–37	4–32	Variable	30 Catfish
L	A	Hydroponic	Researchers, Students	Frequent	19–23	19–23	Basil, Lettuce	-
	B	Aquaponic	Researchers, Students	Frequent	19–23	26–27	Basil, Lettuce	20 Tilapia
T	A	Hydroponic	Public-Access	Constant	21–25	21–25	Basil	-

a Hydroponics refers to a system that produces plants in a liquid media instead of soil. Aquaponics refers to a system that combines aquaculture [raising of fish or other seafood] with hydroponics.

**Table 2.** Summary of microbial results for each of the eight systems samples.

Location	System			Microbial ^a^			

				Total Coliforms		E. coli	

	ID	Type	No. of Samples	No. Below Upper LOD ^b^	Mean (95% CI ^c^) MPN/100-mL in Samples Below Upper LOD	No. Above Lower LOD ^d^	Mean (95% CI ^c^) MPN/100-mL in Samples Above Lower LOD
C	A	Hydroponic	10	1	437.1 (337.2, 555.5)	0	-
	B	Hydroponic	10	1	1986.3 (1222.0, 3300.2)	1	1.0 (0.1, 5.5)
	C	Hydroponic	10	1	1986.3 (1222.0, 3300.2)	1	53.9 (40.5, 69.7)
	D	Aquaponic	10	1	1732.9 (1167.7, 2709.5)	0	-
H	A	Aquaponic	9	7	354.0 (247.0, 524.7)	0	-
L	A	Hydroponic	10	1	1553.1 (1016.2, 2353.1)	0	-
	B	Aquaponic	10	2	1986.3 (1222.0, 3300.2)	0	-
T	A	Hydroponic	10	2	1124.6 (788.1, 1678.9)	1	1.0 (0.1, 5.5)

a One 25-mL sample per system was tested for *Salmonella* presence (No. of Samples = 9), and none were positive.

b Upper limit of detection (LOD) = 2419.6 MPN/100-mL.

c 95% confidence interval. If all samples were above or below the LOD then-was entered.

d Lower LOD = 1 MPN/100-mL.
